# Pressure-assisted sintering and characterization of Nd:YAG ceramic lasers

**DOI:** 10.1038/s41598-021-81194-8

**Published:** 2021-01-15

**Authors:** Avital Wagner, Yekutiel Meshorer, Barak Ratzker, David Sinefeld, Sergey Kalabukhov, Sharone Goldring, Ehud Galun, Nachum Frage

**Affiliations:** 1 Department of Materials Engineering, Ben-Gurion University of the Negev, P.O.B 653, 84105 Beer-Sheva, Israel; 2 Applied Physics Division, Lasers Department, Soreq NRC, 8180000 Yavne, Israel; 3 Department of Applied Physics, Electro-Optics Engineering Faculty, Jerusalem College of Technology, 91160 Jerusalem, Israel; 4 DDR&D IMOD, 61909 Tel Aviv, Israel

**Keywords:** Materials science, Materials for optics, Optics and photonics, Lasers, LEDs and light sources

## Abstract

Spark plasma sintering (SPS) is an advanced one-stage, rapid, near-net shape densification technique combining uniaxial pressure with resistive heating. Various transparent ceramics have been successfully fabricated by SPS, despite the existence of inherent carbon contamination and residual pores. Due to the disk-shape of SPS-processed samples, the technique may be suited for producing thin-disk ceramic laser materials. Nevertheless, an in-depth study of these materials has never been reported. With that goal in mind, the major focus of this study was to characterize the laser performance of Nd:YAG ceramics fabricated by one-stage SPS under conventional (60 MPa) and high (300 MPa) applied pressures. In addition to measuring the lasing slope efficiency and threshold, the passive losses associated with each sample were also evaluated. Surprisingly, it was found that in-line transmittance spectra do not provide accurate predictions of laser performance due to the nature of residual porosity. Moreover, homogeneity and beam quality were assessed, and comparisons were drawn between conventional and high-pressure SPS ceramics. This study lays the groundwork for the future of laser materials fabricated by SPS or similar pressure-assisted techniques.

## Introduction

Most solid-state lasers, other than semiconductor lasers, are based on single crystals, polycrystalline ceramics or glass (as host materials) doped with small amounts of transition metals or rare-earth elements (i.e. active ions). A prominent example of one such laser material is Nd:YAG single crystal. A Nd:YAG laser was first demonstrated as being capable of continuous-wave (CW) operation as early as 1964^1^. Due to its preferable properties, as compared to other materials, Nd:YAG is currently the most widespread and well-known laser material^2,3^. Although single-crystal laser materials have proven to be very useful, they are limited in size, their time of growth is long and expensive, and they display limited flexibility in terms of tailoring specific dopant concentrations. For instance, some dopants (such as Nd^+3^ in YAG) cannot exceed low concentration levels without causing intolerable deformations in the crystal lattice. At the same time, ceramic laser materials offer several advantageous traits over single crystals, including versatile and fast fabrication, heavy and homogeneous doping and allowing for the realization of complicated composite structures. However, the fabrication of high-quality transparent ceramics is more challenging, as compared to single crystals. Indeed, only in 1995 was the first ceramic Nd:YAG laser gain medium demonstrated^4^. In the following years, with improvements in fabrication methods, ceramic laser gain media have been shown to perform similarly to their high-quality single crystal counterparts^5^. The versatility of ceramic fabrication for laser gain media is expected to facilitate the development of new types of advanced laser materials that can only be produced by powder metallurgy methods^6–8^.

For ceramic materials to function as laser gain media, high optical quality with minimal internal scattering and defects is required. The common processing method for highly transparent Nd:YAG ceramics is densification of nano-powders by vacuum sintering^4,5,9–20^. This process is highly effective for producing functional transparent ceramics but requires several time-consuming steps, including preparation of a green body, sintering at a high temperature under high vacuum and post-sintering annealing. More recently, the one-stage pressure- and field-assisted sintering process, known as spark plasma sintering (SPS)^21^, has begun to emerge as a highly effective method for fabricating a variety of transparent ceramics^22–25^. SPS is an advanced sintering technique that combines uniaxial pressure with resistive heating by an electric current. The applied external pressure (typically 50–100 MPa) allows to achieve fully dense samples at relatively low temperatures and after short dwell times. An advanced approach of applying higher pressure during SPS (up to ~ 1 GPa) allows for reduced sintering temperatures while consolidating transparent ceramics with smaller grain size and improved mechanical properties^25–31^. Moreover, dense nanostructured ceramics can be fabricated by this so-called high-pressure SPS (HPSPS) technique, which allows to overcome birefringence in non-cubic structured ceramics^25,27,32^. Due to its potential functionality as a laser gain medium, ceramic Nd:YAG has been fabricated by conventional and high-pressure SPS^33–36^. Nevertheless, fabrication of highly transparent ceramics with sufficient optical quality by SPS is still a challenge, especially reaching the optical quality needed for reliable laser applications.

Like all processing techniques, SPS suffers from inherent drawbacks and limitations. SPS-processed transparent ceramics typically suffer from carbon contamination^37–41^ and relatively high residual porosity (due to the relatively low vacuum of ~ 600 mbar used during SPS, as compared to ~ 10^–5^ mbar used in vacuum sintering). Carbon contamination can be suppressed by sintering additives^22,37^, low heating rates^41–43^ and applying high pressure with advanced tooling made of refractory materials, such as SiC^25,29^ or WC^28^. The residual porosity issue in SPS-processed ceramics is much harder to mitigate. During the final stage of sintering, pores become isolated at triple points and their shrinkage is limited by the pressure of trapped gas within them. The initial pressure in the closed pores depends on the sintering atmosphere, and despite the external pressure applied during SPS, the final size of any residual pores is considerably larger than of pores found following vacuum sintering. By applying high pressure during SPS, it is possible to reduce the residual porosity volume fraction and average pore size^44^.

Despite these drawbacks, SPS offers many advantages, as compared to other fabrication methods. The main advantages of SPS over techniques like vacuum sintering are the one-stage, rapid, near-net-shape production capabilities. This could place SPS at the technological forefront for the fabrication of thin-disk^45–47^ and composite^48^ laser gain media. Since SPS allows to produce samples with predetermined thickness, use of this technique could significantly minimize material waste and expensive post-sintering machining. A few years ago, some laser performance was shown for SPS-processed Y_2_O_3_^49^ and Lu_2_O_3_^50–52^ ceramics. Recently, two-step approaches, such as SPS followed by hot isostatic pressing (HIP)^53^, and one-stage HPSPS^35^ have been shown to produce YAG ceramics with reasonably good laser performance. When comparing conventional and high-pressure SPS processes, the former is easier to implement, with no special tooling requirements and less sample size limitations. Fabrication by SPS can thus elevate the field of laser ceramics to that of ceramic powders in terms of versatility (the latter being very high)^6–8^.

In the present study, transparent polycrystalline Nd:YAG ceramics were fabricated by conventional and high-pressure SPS from synthesized powders. The samples produced by both methods were successfully lased under Ti:Sapphire laser excitation. Comparisons were drawn between the SPS/HPSPS fabrication techniques, Nd concentrations and sample thicknesses. Additionally, the passive losses, homogeneity and laser beam quality of the processed samples were studied. The results are methodically presented, analyzed, and followed by a unified discussion. To the best of our knowledge, this is the first in-depth laser performance characterization of its kind for ceramics fabricated by these techniques. Furthermore, it is the first demonstration of laser oscillations in one-stage SPS-processed doped YAG ceramics.

## Experimental

### Powder synthesis and sample preparation

Nd:YAG powders with various doping concentrations (1, 1.5 and 2 at%Nd) were synthesized using a modified co-precipitation method in which commercial YAG powder (Nanocerox) was doped using Nd nitrate hexahydrate (Nd(NO_3_)_3_·6H_2_O, 99.9%; Alfa Aesar) as a precursor. Since, Nd substitutes for Y in the YAG lattice, the precise YAG stoichiometry was maintained by adding the correct molar ratio of Al nitrate nonahydrate (Al(NO_3_)_3_·9H_2_O, 99%; Alfa Aesar). This process is described in detail in our previous contributions^36,54^ and included lyophilization and subsequent calcination treatments. The powders were consolidated to fabricate single-phase highly transparent ceramics by means of an SPS apparatus (HP-D10 FCT Systeme, Rauenstein, Germany) under conventional (60 MPa) and high-pressure conditions (300 MPa), namely SPS and HPSPS, respectively. Dies with an inner diameter of 20 and 10 mm were used for SPS and HPSPS, respectively. The process parameters (i.e. temperature, pressure, dwell time and heating rate) were also consistent with those previously reported^36^, which were sufficient to ensure full incorporation of Nd ions in the YAG matrix. The SPS-processed samples were later machined to a 10 mm diameter to match the HPSPS-processed samples. Samples with various doping concentrations (1–2 at%) and thickness (0.6–1.8 mm) were fabricated by both methods. All samples were parallel planar polished to an optical grade by Optec Inc. and coated by ELOP (AR@1064 nm s_1_,s_2_ < 1% AOI (angle of incidence) ± 1 deg, HT@800–890 nm cs_1_,s_2_ > 97% AOI ± 1 deg).

### Sample characterization

In-line transmittance spectra for the samples at wavelengths of 200–1100 nm were obtained using a UV–VIS spectrophotometer (V-530; JASCO). A scanning electron microscope (ESEM, Quanta 200, FEI) was used to image the microstructure of sample fracture surfaces. A trasnmission electron microscope (TEM, T-12 Tecnai, FEI) was used to image pores present in HPSPS-processed sample thin lamella prepared by a focused ion beam (FIB, Helios-G4 UC DuelBeam system, FEI). A confocal laser scanning microscope (CLSM, Zeiss LSM880, Oberkochen, Germany) was used to characterize residual porosity content, similar to the analysis done by^44,55,56^. A Z-stack 3D image (75 × 75 × 20 µm^3^, with a distance of 130 nm between slices) was acquired in reflection mode using a 458 nm argon laser and an Airyscan (Zeiss, Oberkochen, Germany) detector. The detection limit of this analysis is approximately ~ 50 nm. The results were analyzed with the Fiji ImageJ software package using the “Threshold” function.

### Laser oscillator characterization methods

**Figure 1:**
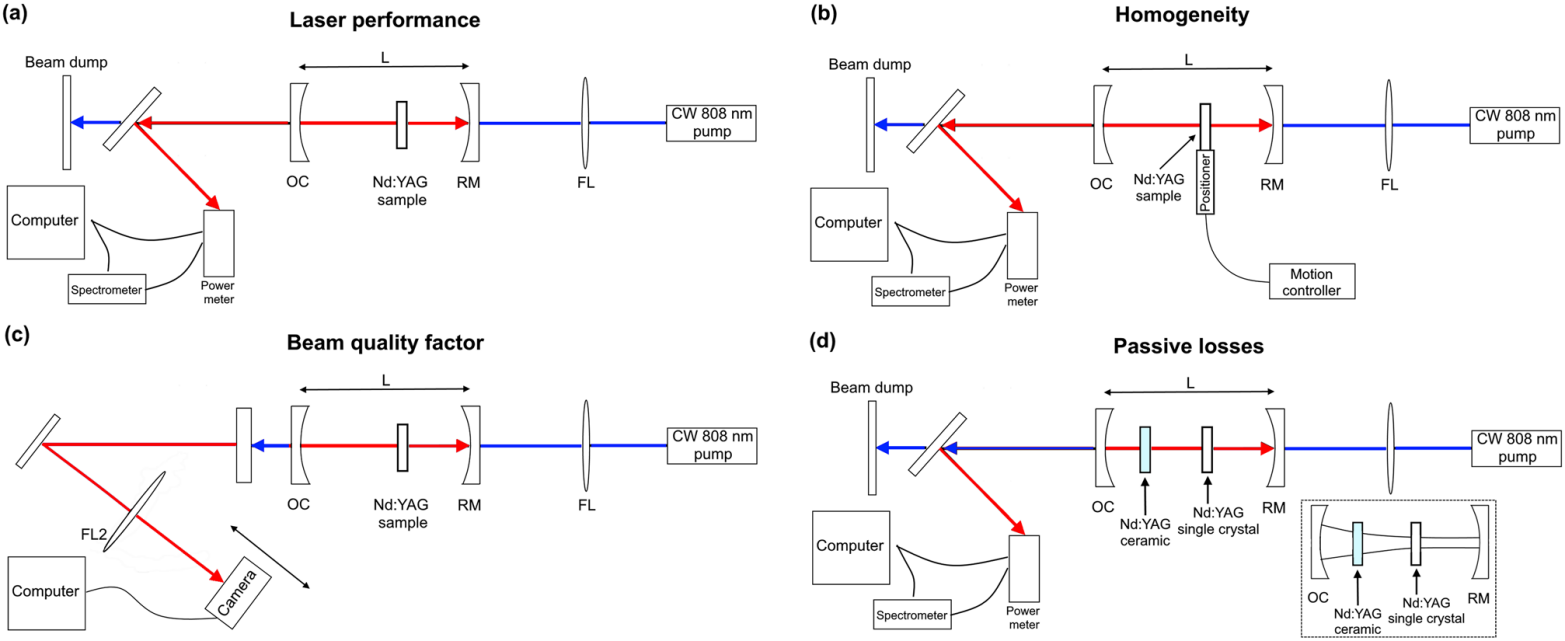
Schematic of the optical setup used for characterization of (**a**) laser performance (efficiency and threshold), (**b**) homogeneity and (**c**) M^2^ beam quality factor (**d**) passive losses.

Laser performance, lasing slope efficiency and threshold were evaluated using the set-up described in Fig. 1a. A tunable CW Ti:Sapphire laser was set to 808 nm and used as the pump. Samples were placed at the waist of the optical resonator, which was composed of a dichroic rear mirror, highly reflective at 1064 nm and highly transitive at the pump wavelength, and an output coupler mirror, partially reflective at the lasing wavelength, 1064 nm. The pump laser Rayleigh range was about 10 mm, thus eliminating sensitivity to sample thickness and exact placement. Output coupler mirrors with 70–95% reflection were exchanged in compliance with the different experiments. Laser emission was measured by an Ophir power meter, and wavelengths were measured by an Ocean optics spectrometer. To evaluate the optical lasing setup, an additional commercial single crystal Nd:YAG sample (Union Carbide Corporation) was evaluated and served as a reference measurement.

To evaluate the homogeneity of the SPS- and HPSPS- processed samples, we scanned two samples along their diameter using a lateral scanning motor inside the lasing cavity (Fig. 1b). The laser performance was measured along the lateral path at constant speed at regular intervals. The SPS and HPSPS samples were of similar thickness (0.9 mm) and doping concentration (1.5%). Three different pump power values were used, and laser emission was measured along the scanning route. Thereafter, lasing efficiency and threshold could be calculated along the sample diameter.

The laser output beam quality factor (M^2^) was evaluated and compared to what was obtained upon lasing with the commercial crystal. This was assessed by focusing the laser beam, measuring its diameter around the waist (using an Ophir optronic beam profiler and BeamGage software) and analyzing the results (Fig. 1c)^57^.

Finally, to measure the losses associated with each sample the following method was used. First, the Findlay-Clay^58^ approach was used to calculate the nominal round-trip losses (L) inside a laser cavity; the commercial single crystal was placed at the waist of the optical resonator and the output coupler mirror the laser slope efficiency (σ_s_) and threshold (P_th_) were measured with 70%, 80%, 90% and 95% reflection mirrors in turn. Following this analysis, the intersection with the y axis is equal to L which was found to be 0.048. The next step was placing a ceramic SPS or HPSPS sample between the single crystal and the 95% output coupler mirror (Fig. 1d). Then, the new laser threshold (P’_th_) was measured to calculate passive losses (ΔL) introduced by the ceramic sample.

## Results

### Microstructural characterization

**Figure 2:**
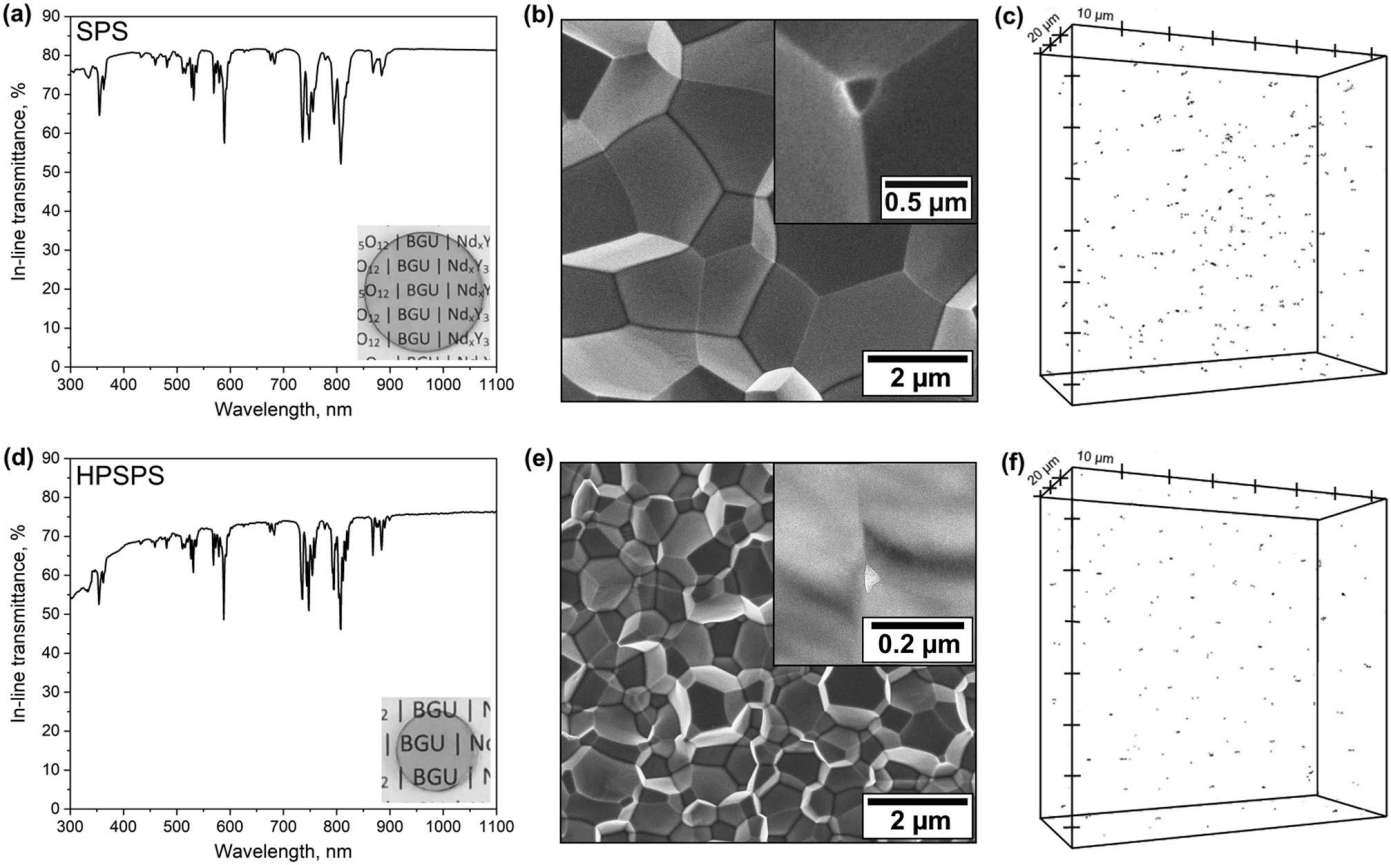
SPS-processed 1.5 at% Nd:YAG (**a**) in-line transmittance spectrum, sample appearance insert, (**b**) SEM image of fracture surface microstructure, close-up on pore in top insert and (**c**) porosity distribution obtained by CLSM in reflection mode. The same follows for the HPSPS-processed 1.5 at% Nd:YAG sample (**d**–**f**) with insert in (**e**) being a TEM image.

SPS- and HPSPS-processed Nd:YAG ceramic samples were compared in terms of appearance, microstructure and residual porosity (Fig. 2 shows 1.5 at% Nd:YAG as an example). The effect of Nd doping concentration on optical properties was also reported in detail in a previous contribution^36^. Furthermore, the microstructure of the SPS- and HPSPS-processed samples exhibited a significant difference in grain size and residual porosity (Fig. 2c, d). The lower temperature, shorter dwell time and high pressure applied during HPSPS suppressed grain growth, giving rise to finer microstructure. The residual porosity in the samples was characterized by confocal laser scanning microscope (CLSM) (Fig. 2e, f) and yielded similar results to those presented previously^44^. It should be noted the microstructure and porosity characteristics are determined by the sintering regime; therefore, all the samples fabricated under the same conditions (SPS or HPSPS) had no detectable difference in these characteristics. There are fewer residual pores detected in HPSPS-processed samples and they are smaller than those in the SPS-processed samples due to the high-pressure used during sintering. Since pores exist at grain boundaries and mostly triple points, the number of pores in HPSPS samples is probably greater and more frequent (the size of many of them is smaller than the detection limit of the CLSM). It is important to remember that only pores greater than ~ 50 nm are detected in the CLSM analysis (Fig. 2). Interestingly, the CLSM analysis makes it possible to discern some grain morphology in the SPS-processed samples due to the relatively large pores (~ 150–300 nm, as compared to ~ 30–100 nm in HPSPS) and grains (~ 5 µm, as compared to ~ 1 µm in HPSPS). In the HPSPS-processed sample, many pores are sized below the detection limit of the microscope and, therefore, clear grain layouts cannot be seen.

### Laser efficiency and threshold

**Figure 3:**
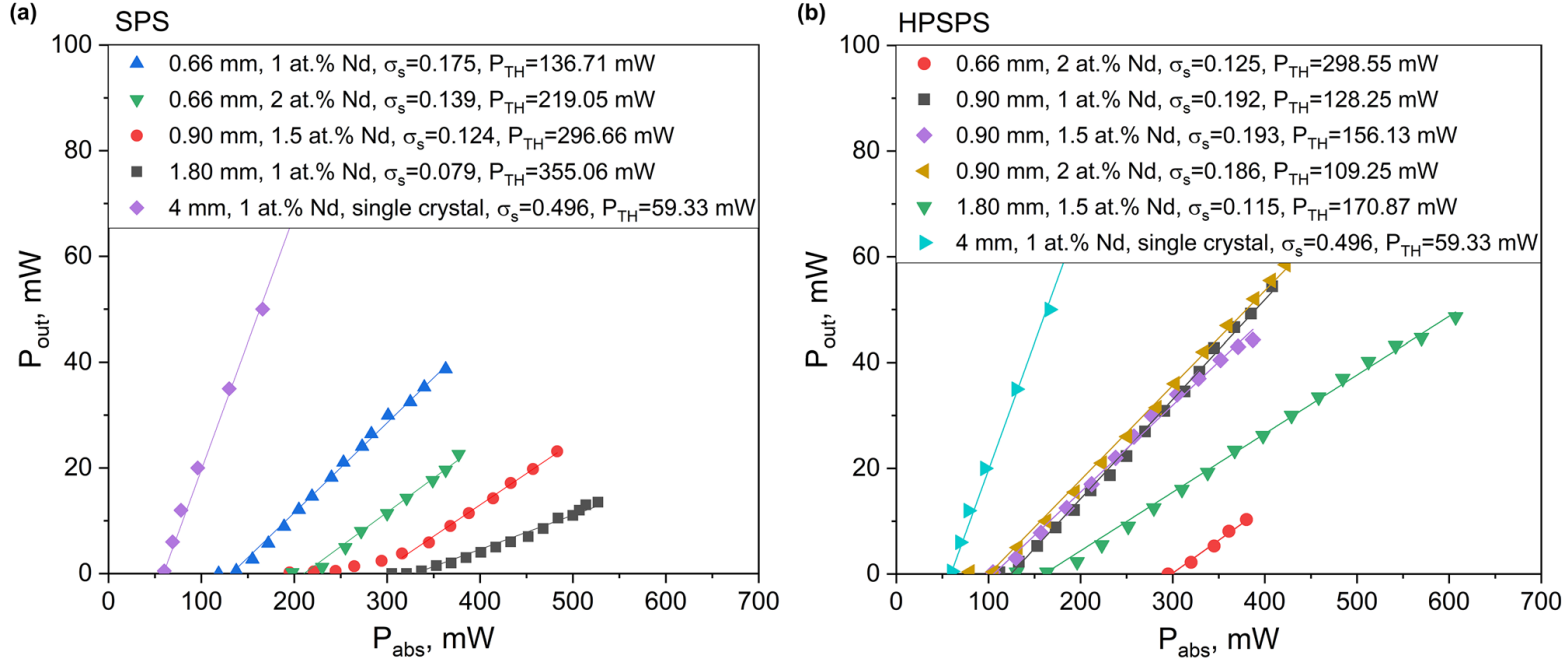
Laser efficiency slopes for (**a**) SPS- and (**b**) HPSPS-processed samples, including results for a commercial single crystal shown for comparison.

Our next step involved evaluating and comparing the laser performance of all SPS- and HPSPS-processed samples. A schematic of the optical setup is shown in Fig. 1a, with each sample being placed in turn at the denoted position. The laser output power versus the absorbed power (i.e. efficiency slopes) for the various ceramics is presented in Fig. 3. In addition, the threshold power (P_TH_) and lasing slope efficiency (σ_s_) values were calculated and are presented in the legend. It was observed that lasing performance varied at difference lateral positions on the samples and it should be noted that the presented values were measured at good lateral positions found for each sample (“sweet spots”). This indicates that while the samples appear visibly homogenous (Fig. 2), when considering lasing performance that is apparently not the case. We elaborate on this in the following section. From the presented results, it is clear the efficiencies of these ceramics are lower than those of their commercial single-crystal counterparts. Furthermore, the laser performance measurements showed the HPSPS-processed samples to have better performance overall with higher laser efficiencies, lower threshold and higher beam quality. With that being said, our results for SPS-processed Nd:YAG are on par or superior to those reported for other SPS-processed ceramic gain media^49–53^. It should also be noted that it was significantly easier to get the HPSPS-processed samples to lase compared to SPS-processed samples, requiring less fine-tuning of the resonator (similar to the commercial single crystal). To the best of our knowledge, this is the first report of laser performance for doped YAG ceramics achieved by one-stage SPS process with no post-sintering treatment (such as HIP^53^).

Another observation from the results is the little, if any, dependence on doping concentration in the range measured here (1–2 at%Nd). The 0.9 mm-long HPSPS-processed samples with doping concentrations of 1, 1.5 and 2 at% Nd displayed similar laser performance. It should be noted, that at least in the naive form of the 4-level laser equations for CW operation^3^, the doping concentration should not affect the laser slop efficiency (in terms of absorbed pump to emitted laser light). This is true so long as the doping concentration does not effectively contribute to the scattering losses, and the so called “dead sites” in which neighboring ions quench so fast as to alter the effective quantum efficiency. The laser threshold however should depend upon the doping concentrations; The threshold is explicitly dependent upon the fluorescence lifetime which is shortened due to concentration quenching as the active ion concentration is increased^36^. However, the laser threshold (as well as the slope efficiency) is strongly dependent upon the scattering losses. Since the scattering losses in the experiments presented above are quite large, the contribution of the fluorescence lifetime variation due to concentration quenching is relatively small. This may explain the similar results acquired with the HPSPS ceramics of identical thickness but different doping concentrations.

### Homogeneity

**Figure 4:**
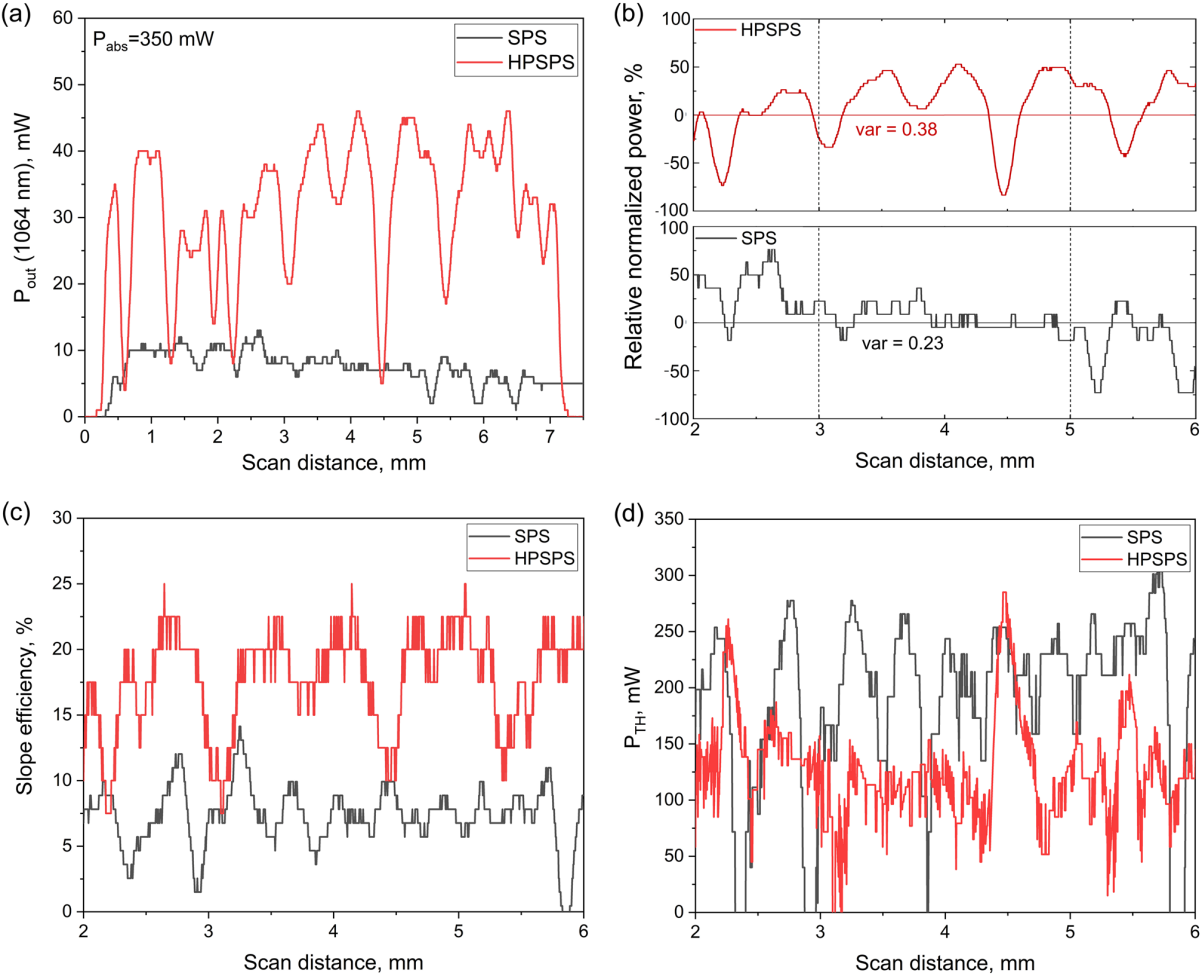
Plots for 0.9 mm, 1.5 at% Nd SPS- (black) and HPSPS-(red) processed samples, a comparison of (**a**) output power for the same absorbed power, (**b**) relative normalized power, calculated from three output power versus absorbed power plots, (**c**) slope efficiency and (**d**) threshold power.

Another important issue when dealing with ceramics is the inhomogeneity that can exist in sintered samples. Such inhomogeneities can lead to significant differences in laser power outputs of ceramic lasers. The microstructure imaging presented above shows consistency at the microscale. Yet, while manually scanning the ceramics for the best spot to measure laser performance, it was evident that there were significant variations within the macroscale of each sample. To study this macro inhomogeneity more carefully, samples were mounted on a mobile stage which moved along the sample diameter at a constant speed, while measuring the laser output power at regular intervals (setup shown in Fig. 1b). The two samples chosen to demonstrate this analysis were both 0.9 mm in length with 1.5 at% Nd doping concentration, with one being SPS- and the other being HPSPS-processed. By performing this measurement at a few different absorbed power values, it was possible to calculate the threshold and slope efficiency at each point along the diameter of the sample (Fig. 4). Several comparisons were made between the SPS- and HPSPS-processed samples. Initially, the output power for the same value of absorbed power was assessed (Fig. 4a), and then the normalized power (Fig. 4b). This was done so as to appreciate the variance of the output power for the two samples. Finally, the slope efficiency (Fig. 4c) and threshold power (Fig. 4d) were compared. It is evident that HPSPS-processed samples generally exhibit higher laser output than do SPS-processed samples, but also present comparably larger variance with a few points having efficiencies as low as SPS-processed samples. These observations of inhomogeneity in laser performance are considerable and should be addressed.

Typically, microstructural inhomogeneity found in materials processed by SPS is predominantly related to the radial direction due to possible radial temperature and stress gradients^59^. Contrary to this, here we show that there is a degree of inhomogeneity (to which lasing performance is very sensitive) that does not necessarily correspond to a certain radial position. It can be observed SPS-processed samples show some radial dependency in the variance of output power, showing lower variance in the central part (~ 2 mm) of the sample (Fig. 4b). Whereas, in HPSPS-processed samples the variance of output power is high across the whole sample diameter. Therefore, it stands to reason this inhomogeneity is affected by the pressure applied during sintering, which could result is slight local variations in porosity characteristics (size and distribution). These findings are important since it revealed a macro-level of inhomogeneity in SPS/HPSPS-processed samples (with otherwise homogenous appearance) that could not have been detected otherwise. This subject must be investigated further. To the best of our knowledge, this is the first time such a systematic laser performance homogeneity measurement was performed on such laser ceramics.

Two important observations regarding inhomogeneity were made: (a) It does not correlate to radial position as would be expected after SPS, and (b) that it is much more substantial in HPSPS compared to SPS. We hypothesize this inhomogeneity arises from very slight variations in the residual porosity. Since the main difference between SPS and HPSPS is the applied pressure, it would be reasonable to deduce that this inhomogeneity is strongly related to this factor. Consequently, we propose that this inhomogeneity could be remedied by adjusting the pressure and temperature regimes during sintering. For instance, studies on fabrication of ceramics from nano-powders by SPS demonstrated high sensitivity of the obtained microstructure to the pressure regime (i.e. loading schedule and rate, pressure stages and maximum pressure)^60–62^.

### Beam quality factor

**Figure 5:**
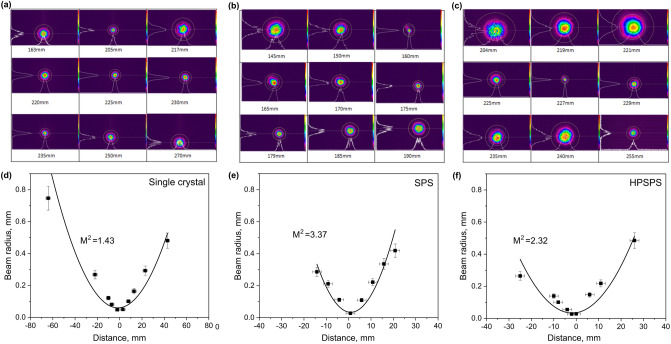
M^2^ measurements: a series of images taken at different locations before and after the waist location for (**a**) the reference crystal, (**b**) an SPS-processed sample, and (**c**) a HPSPS-processed sample. Results of beam diameter versus distance, yielding different M^2^ values for (**d**) the reference crystal, (**e**) an SPS-processed sample, and (**f**) a HPSPS-processed sample.

The beam quality factor M^2^ is important for many laser applications. M^2^ is the factor that defines the quality of the laser beam by comparing its divergence from an ideal, single mode Gaussian beam for which M^2^ equals 1^56^. A schematic of the setup used for this measurement is presented in Fig. 1c. For these measurements, the commercial single crystal was used, together with the two samples used for the homogeneity analysis (0.9 mm in length, 1.5 at% Nd, one SPS-processed and the other HPSPS-processed). The camera placed near the beam waist was moved along the optical axis. Ten pictures of the beam were used to measure beam diameter at each point and fit the results to define M^2^^56^. It should be noted the effective resonator length was slightly changed for the measurement of the commercial single crystal sample since it is 4 mm long, as compared with the 0.9 mm-long SPS- and HPSPS-processed ceramics. From the results presented in Fig. 5, the beam quality of the commercial single crystal was highest (M^2^ = 1.43), followed by that of the HPSPS- (M^2^ = 2.32) and SPS- (M^2^ = 3.37) processed ceramics. The HPSPS-processed sample to has higher beam quality (smaller M^2^) compared to the SPS-processed sample which is indicative of less scattering.

### Passive losses

Total laser efficiency depends upon various parameters. These parameters can be roughly divided into the following categories: a. pump efficiency, namely, the fraction of the pump photons that excite the lasing ions; b. laser emission efficiency, namely, the fraction of exited ions that contribute to the laser emission; and c. the cavity extraction efficiency, namely, the fraction of emitted laser photons that leave the cavity without being lost (scattered or absorbed by impurities). A crucial factor reflecting the quality of a laser material as a host is the passive losses inflicted by the laser light. Even very small variations in such losses may be crucial since the lasing photons bounce back and forth in the cavity and thus accumulate losses proportional to the average number of such round trips. It is thus beneficial to evaluate and compare passive losses induced by the different ceramic samples. This should also clarify the difference in laser performance presented in the previous section. Firstly, the total nominal losses associated with the optical resonator were evaluated using the Findlay-Clay approach^3, 58^. Then, the passive losses of each ceramic sample were studied using the setup in Fig. 1d and the following analysis. Since the threshold power is proportional to the sum of transmittance of the output coupler mirror ($T$) and the resonator optical losses ($L$) (including the intra-crystal losses) calculated by the Findlay-Clay approach (Eq. (1)), the threshold measured with the ceramic sample has an added component of the passive losses associated with that sample (${\Delta }L$) (Eq. (2)).

1 $$P_{TH} \propto \left( {T + L} \right)$$

2 $$P_{TH}^{^{\prime}} \propto \left( {T + L + {\Delta }L} \right)$$

By dividing Eq. (1) by Eq. (2), it is possible to reach a comprehensive equation for calculating passive losses (Eq. (3)).

3 $${\Delta }L = \left( {\frac{{P_{TH}^{^{\prime}} }}{{P_{TH} }} - 1} \right)\left( {T + L} \right)$$

In the equations, $P_{TH}$ and $P^{\prime}_{TH}$ are the laser threshold power without and with the ceramic sample, respectively. It should be stressed that L represents the fractional cavity round-trip losses and thus is dimensionless. ${\Delta }L$ can be expressed in terms of a loss coefficient, $\alpha$ (loss per unit length) as depicted in Eq. (4), with $x$ denoting the sample length. The boundary conditions for the exponential correlation between the losses and absorption coefficient (Eq. (4)) are ${\Delta }L = 0$ at a sample length of 0 mm and ${\Delta }L = 1$ at an infinitely large length.

4 $${\Delta }L = 1 - {\text{exp}}\left( { - \alpha x} \right)$$

**Figure 6:**
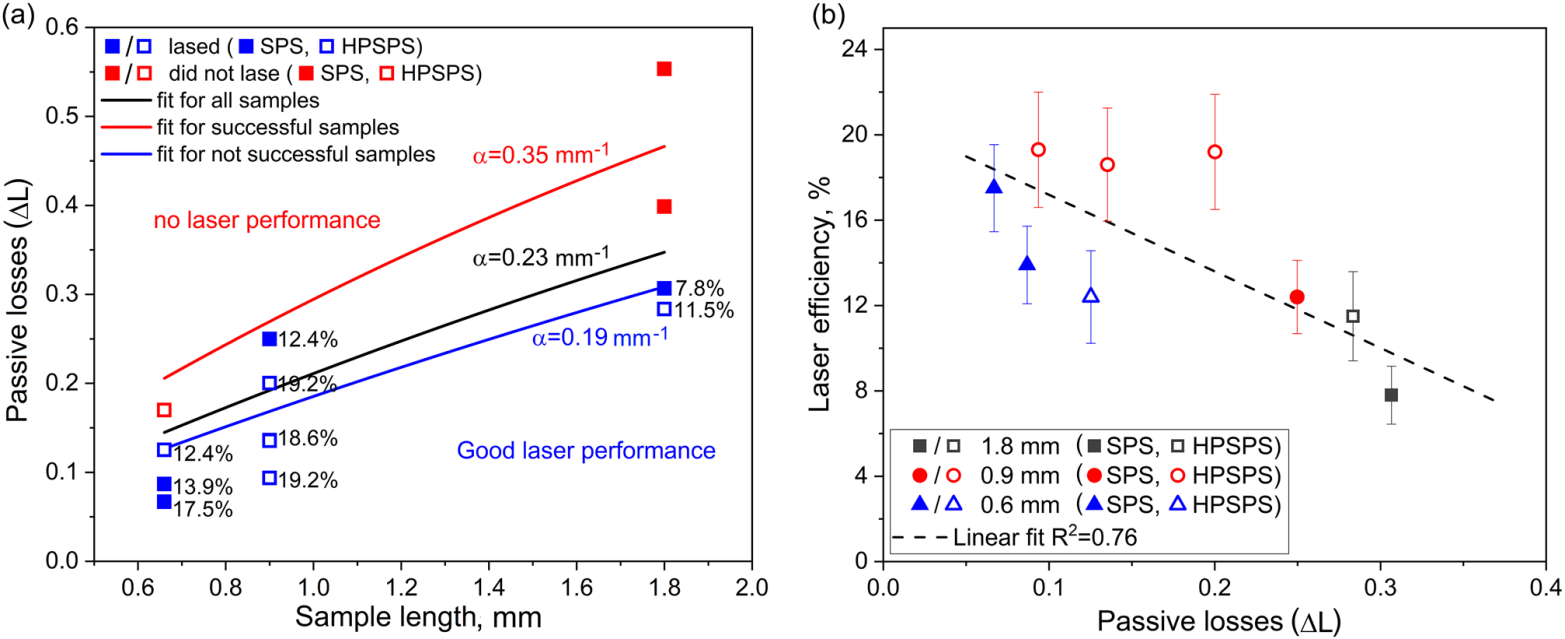
(**a**) Passive losses as a function of ceramics length. Lasing efficiencies are noted for each sample. Blue and red represent samples that lased and sample that did not laser, respectively. (**b**) Slope efficiency as a function of passive losses for samples which lased. Different colors account for different sample lengths. Full and hollow notations represent SPS- and HPSPS-processed samples, respectively.

Figure 6a displays the calculated sample losses (${\Delta }L$) as a function of sample length. Three exponential fitting curves are presented, with the black curve fitting all samples, the blue curve fitting samples that displayed laser performance and the red curve fitting the sample which did not. Full and hollow square notations were used for SPS and HPSPS, respectively. It is possible to discern conditions for good laser performance, namely, when the impurity absorption/scattering-losses coefficient (α_loss_) is below 0.19 mm^−1^. Furthermore, it is possible to see that laser oscillations were not successfully achieved for samples with α_loss_ above 0.35 mm^−1^. Between these two conditions, there is a mid-range within which are found both samples that had discernable laser performance and those which did not. Generally speaking, for any given length, the lasing efficiency was inversely proportional to the passive losses. Figure 6b shows the laser slope efficiency as a function of passive losses for samples which lased. The standard deviation was calculated according to the variance measured in the homogeneity section. A clear correlation be discerned, where smaller losses are related to higher efficiency, as expected. Moreover, it was possible to discern a trend where at the shortest length SPS-processed samples have lower losses compared to HPSPS and at longer lengths it was reverse.

The passive losses associated with each sample allowed us to predict laser efficiency without having to actually lase the sample, and form conclusions about the factors affecting laser performance in ceramics fabricated by pressure-assisted sintering techniques.

## Discussion

Light scattering is the key factor for determining functionality of transparent ceramics in general, and even more so for ceramic laser gain media^5,63^. Scattering occurs at the interface between regions with different refractive indices. In cubic structured ceramics, such interfaces usually exist between a ceramic matrix and pores or second phases, or between regions of the matrix with variations of stress^63^. Mie theory defines the dependence of light scattering on the size of the scattering center, relative to the wavelength^64,65^. Since residual porosity in ceramics results directly from powder metallurgy fabrication methods, it can never be entirely suppressed. As for grain boundaries, their small thickness (< ~ 5 nm) means their contribution to visible and IR light scattering is negligible. Therefore, inherent residual porosity is the most critical microstructural feature which causes detrimental scattering centers in single phase cubic structured ceramics, such as YAG. In a laser system, the emission wavelength passes through the gain medium material multiple times before exiting the optical resonator. In every pass, there are losses which decrease the gain and reduce the efficiency of the system. Thus, scattering from pores in ceramic lasers increases the laser threshold and decreases efficiency significantly, as compared to their single crystal counterparts. As seen in our results, some samples displayed adequate laser performance, while others simply did not achieve sufficient gain (Fig. 3). We thus tried to understand what differentiated these samples.

The simplest measure of scattering losses in a sample is transmittance measurements. We have shown here and previously^36,54^ that the in-line transmittance spectra (detecting light scattered at angles smaller than ~ 1°) of SPS samples are higher compared to HPSPS. It was established that Mie scattering (forward directional) dominates in SPS-processed samples and Rayleigh scattering (random direction) dominates in HPSPS-processed samples. Therefore, it should stand to reason that their laser performance too should be better. However, our results show this is not the case. The SPS-processed samples had higher in-line transmittance as compared to HPSPS-processed ones (for same length and doping concentration), though the laser performance of the latter was considerably better. An example of this can be seen for SPS- and HPSPS-processed samples of 0.9 mm in length and 1.5 at% Nd (in-line transmittance spectra in Fig. 2a, d and laser performance Fig. 3a, b).

**Figure 7:**
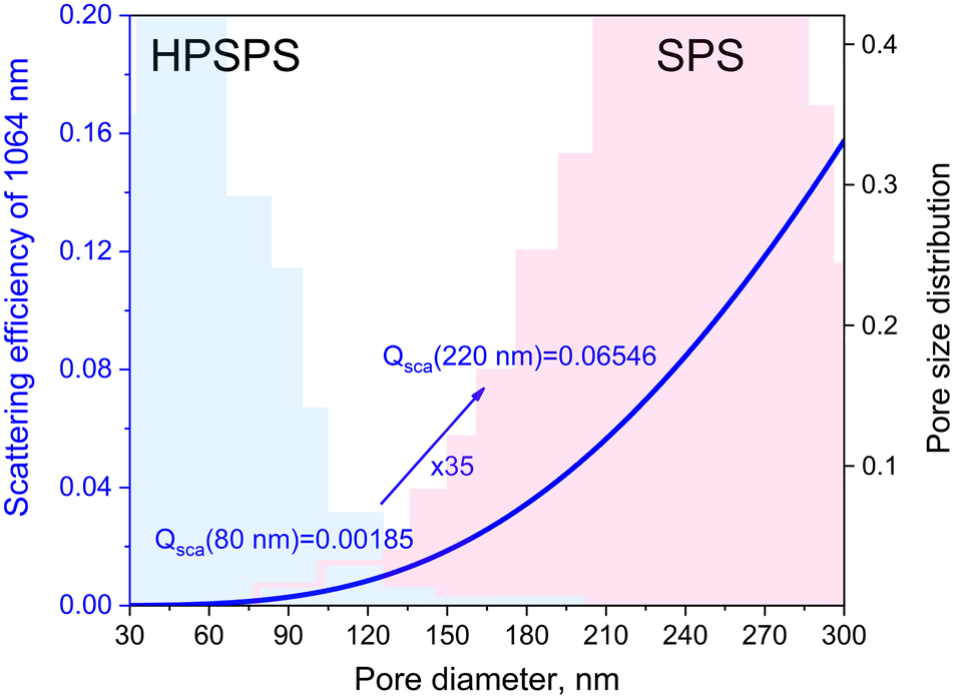
Scattering efficiency as a function of pore diameter calculated using MiePlot^68^, the shaded light blue area represents the pore size distribution in HPSPS samples (gamma distribution according to^69^) and the shaded pink area represents the same for SPS samples. It is possible to see the scattering efficiency is 35 times higher for average pores in the SPS samples compared to those is HPSPS.

Seemingly, the in-line transmittance measurement is more forgiving for two reasons: (1) the impinging light only passes through the sample once, and (2) some forward scattered light will still reach the detector and count in favor of high in-line transmission. Meanwhile, in the optical resonator the beam travels many times through the sample and all scattering events compete with achieving gain by stimulated emission. Therefore, we should only account for dependence of the scattering efficiency on the pore size and discount its directionality. From Mie theory calculations, the scattering efficiency from pores of ~ 200 nm (average pore size in SPS) is more than 30 times higher than from pores of ~ 80 nm^66–68^ (Fig. 7). Accordingly, even if the beam encounters ten times the number of pores in HPSPS samples, the scattering in SPS samples will still be greater. This understanding is very important since the common assumption for ceramic laser materials is that higher in-line transmittance would result in better laser performance. We have established that this is not necessarily always the case, porosity volume fraction, pore size and distribution must all be considered. It is important to note, within each set of samples (SPS or HPSPS) the in-line transmittance does correlate with the laser efficiency. However, this correlation does not carry between the sets because the porosity characteristics are very different (due to the different sintering regimes). Since vacuum sintering (resulting in negligible porosity and minute pore sizes) was used to fabricate most of the ceramic lasers demonstrated in the literature this phenomenon was not widely recognized.

To try and correlate the laser efficiency with losses by a different means, we performed the described passive losses analysis. In this case, since the measurement was performed by placing the ceramics inside the optical resonator, a good correlation was achieved (Fig. 6b). The beam quality measurements (Fig. 5) also support the explanation of less scattering in HPSPS samples according to Li et al.^70^ who investigated the effect of scattering on the beam quality. Consequently, short sample length SPS-processed samples perform better due to the smaller probability of encountering a pore. Whereas, in long lengths the chances of scattering become higher and the larger pores are more detrimental to the laser performance compared to the smaller HPSPS ones. This is the same physical reason why ceramics with any degree of porosity lose transparency with increasing thickness/length (Beer-Lambert law)^71^.

**Figure 8:**
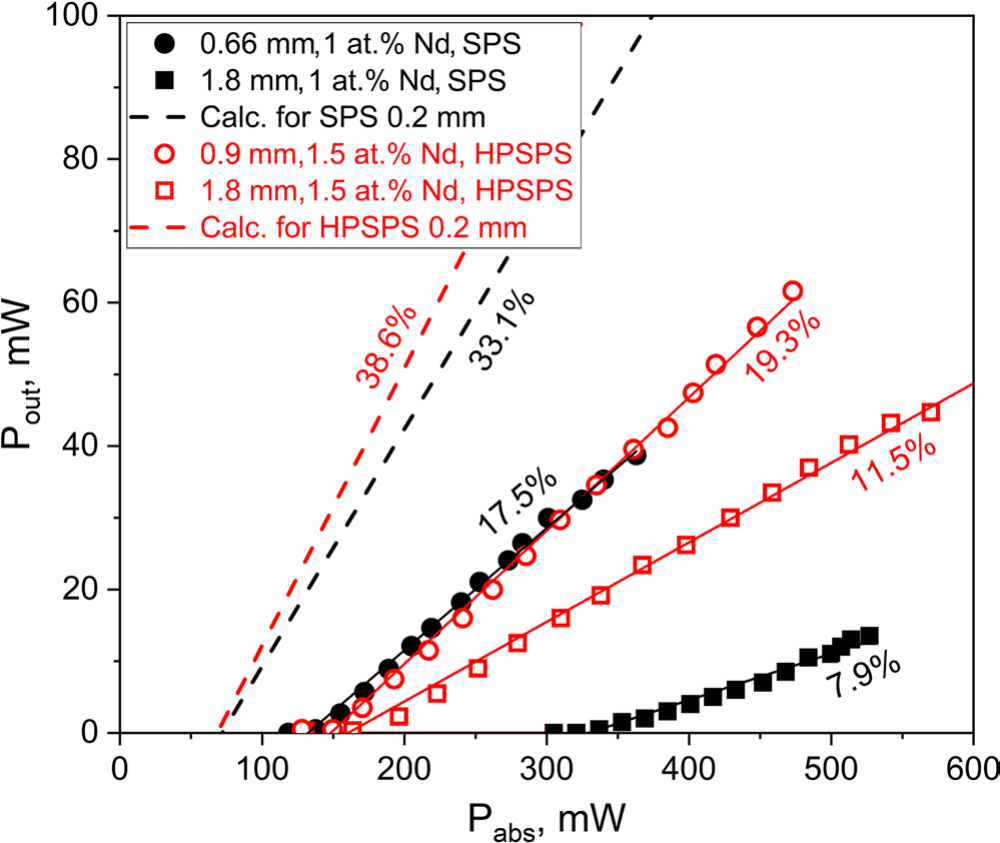
Theoretical estimation of laser output power versus absorbed power, showing the effect of ceramics length on the efficiency of 1 at% Nd SPS-processed Nd:YAG and 1.5 at% Nd HPSPS-processed ceramics.

There is no denying the laser efficiencies of SPS-processed Nd:YAG ceramics are still lower than those of high-quality ceramic samples fabricated by vacuum sintering and/or hot isostatic pressing. This makes sense, since these technologies have been studied for laser applications for over twenty years, while SPS is a less mature technique in that regard. Moreover, ceramic laser materials doped with elements having longer emission wavelengths (e.g. Er, Tm, Ho) will be less susceptible to residual pore scattering and may be the future direction of SPS-processed ceramics^66^. We propose that pressure-assisted sintering techniques (such as SPS) could be an effective and cost-efficient method for fabricating ceramic materials for a thin-disk laser design. Since it combines the advantage of near net-shape fabrication of flat disks using minimal raw material and post-processing machining, together with reduced passive losses due to inherent residual porosity. Furthermore, thin disk configurations allow design of improved cooling systems, which reduce thermal effects and enable higher dopant concentration. This is very useful since highly doped gain media have reduced thermal conductivity, making them difficult to use in standard laser configurations. It is possible to estimate potential performance based on our experimental results. After calculating average losses of samples that showed reasonable laser performance, laser efficiency and threshold could be also calculated for thinner samples^3^. The calculated plots for samples prepared by both SPS and HPSPS are presented in Fig. 8. It can be seen that SPS- and HPSPS-processed Nd:YAG could achieve efficiencies of 33% and 38%, respectively, for 200 µm-thick samples. This is encouraging since SPS is a more practical fabrication method for production of thin-disk laser gain media, due to simple and cheaper graphite tooling with fewer size limitations. It should be noted that on average for our samples in such configuration the pump beam should make 10 round trips (back and forth) through the gain medium in order to achieve over 98% absorption. Even though the pump beam is generally less sensitive to scattering than the laser beam, scattering losses will reduce the available pump power. Assuming similar scattering loss coefficient for the pump beam as the one measured for the laser wavelength; it is calculated that 10 round trips through the gain medium will sum-up to a total of about 4% pump power lost. This is reasonable and solidifies our conceptualization of thin-disk lasers based on SPS-processed ceramics.

Future work should focus on the optimization of conventional and high-pressure SPS process parameters for laser materials. The understanding and lessening of the reported inhomogeneity and reducing average pore sizes should be the top priorities. Therefore, a wide variety of sintering temperatures, applied pressure regimes, dwell times and heating rates should be considered. Additional optimization of the resonator cavity to match thin-disk samples can also be attempted. Furthermore, our powder synthesis method and sintering process could be easily adapted for other dopants and laser materials.

## Conclusions

This contribution presents the first in-depth optical characterization of doped YAG ceramics fabricated by conventional and high-pressure SPS. These pressure-assisted sintering techniques provide rapid near-net shape densification, albeit with residual porosity that affects laser performance. SPS- and HPSPS-processed Nd:YAG ceramic samples with different lengths and doping concentrations were analyzed and compared to a commercial single crystal laser gain medium. Laser performance, homogeneity and beam quality were evaluated. To the best of our knowledge, this is the first report showing laser operation in conventional SPS-processed doped YAG ceramics with no additional post-sintering treatment. The passive losses were studied in a unique and relatively simple manner. Macro inhomogeneity in laser performance was shown systematically for the first time and highlights another research avenue to be perused. Our work further proves that SPS can produce transparent laser ceramics of high optical quality, solidifying SPS as an excellent research tool for such materials. The combination of optical quality, speed, versatility and near net-shape fabrication allows SPS to be used for statistical work and exploring new laser materials. These research findings provide the first step towards understanding the problems facing SPS-processed ceramics for laser applications, and optimization is an ongoing effort in the science community. This study lays the groundwork for the future of thin-disk laser materials fabricated by SPS/HPSPS or similar pressure-assisted methods.

## Data Availability

All data needed to evaluate the conclusions in the paper are present in the paper. The raw/processed data required to reproduce these findings can be shared from the corresponding authors upon reasonable requests.
